# Cytotoxic, Antioxidant, and Metabolic Enzyme Inhibitory Activities of *Euphorbia cyparissias* Extracts

**DOI:** 10.1155/2020/9835167

**Published:** 2020-10-29

**Authors:** Mona Alonazi, Habib Horchani, Mona Alwhibi, Abir Ben Bacha

**Affiliations:** ^1^Biochemistry Department, Science College, King Saud University, Riyadh, Saudi Arabia; ^2^Science Department, College of Rivière-Du-Loup, Rivière-Du-Loup, Québec, Canada G5R1E2; ^3^Botany and Microbiology Department, Science College, King Saud University, Riyadh, Saudi Arabia; ^4^Laboratory of Plant Biotechnology Applied to Crop Improvement, Faculty of Science of Sfax, University of Sfax, Tunisia

## Abstract

Plants of the Euphorbia genus present a wide range of therapeutic applications. This study is aimed at investigating new antidigestive enzyme agents from *Euphorbia cyparissias* through inhibition of lipid and carbohydrate absorption, to evaluate their potential applications for the treatment of metabolic syndrome. Lipase, phospholipase, protease, *α*-amylase, *β*-glucosidase, and xanthine oxidase activities under treatment with aqueous and ethanolic extracts of *Euphorbia cyparissias* were observed to evaluate the inhibitory effect of these extracts, as well as their antioxidant and cytotoxic effects. Results showed that ethanolic and aqueous extracts exhibited important inhibitory activity in a concentration-related manner on digestive enzymes, which is more effective than the commercial drugs used as controls. Results also showed that, out of the two extracts tested, the ethanolic extract presented the most promising results in inhibiting the activities of all digestive enzymes used. Moreover, the two extracts displayed a higher reducing power than that of the positive control used. The obtained results, together with previous reports in the literature, strongly suggest that *Euphorbia cyparissias* extracts may be natural inhibitors of the digestive enzymes and thus a potential new drug for metabolic syndrome treatment.

## 1. Introduction

Metabolic syndrome is a serious and escalating worldwide public health threat that directly increases the risk of developing obesity, diabetes, atherosclerosis, and hypertension, or combinations [1]. These metabolic abnormalities are related to, among other enzymes, lipolytic enzymes such as pancreatic lipases, phospholipases, and proteases and metabolic enzymes like *β*-glucosidase, *α*-amylase, and xanthine oxidase (XO) [2]. The prevalence of the metabolic syndrome is increasing to epidemic proportions not only in the United States and the remainder of the urbanized world but also in developing nations [3]. In most countries, between 20% and 30% of the adult population suffer from metabolic syndrome [4]. Because of this high prevalence and the consequences of metabolic syndrome, which is an important contributor to the increase in cardiovascular and diabetes risks worldwide, special therapeutic attention should be devoted.

The currently used approach is focused on weight reduction and physical activity. However, it has been reported that polyphenolic extracts from various plants may positively influence different features of metabolic syndrome and control hyperlipidemia in people with obesity, by directly influencing the activities of metabolic enzymes [5, 6]. Independent of their antioxidant activities, these extracts were found to be effective inhibitors of lipases [7], proteases [8], *β*-glucosidase [9], and *α*-amylase activities [10] and can also cause insulin-like effects in glucose utilization [11]. While multiple types of drugs have been developed to help ease the pain, they do not come without side effects. Indeed, anti-inflammatory drugs such as Naproxen, Celecoxib, and Indomethacin are harmful for the digestive system and can lead to stomach pain, diarrhea, nausea, and stomach ulcers [12]. Alternatively, plants and their products have been used as drugs for thousands of years, and the recently renewed interest in researching effective drugs with less adverse effects has provided a definitive stimulus for the development of natural product chemistry.

Some *Euphorbia* plants have been identified as having antioxidant effects, such as *Euphorbia royleana Boiss* [13], *Euphorbia hirta* L. [14], *Euphorbia heterophylla* Desf. [15], and *Euphorbia cuneata* Vahi [16]. Interestingly, the use of Euphorbia spp. L constituents as remedy for diseases, like antipyretic, analgesic, anxiolytic, and anti-inflammatory, has been previously stated in literatures [14] and thus can be used to inhibit secreted inflammatory enzymes such as secreted phospholipase A2 (PLA2) [17], among other phospholipases. According to several studies, digestive enzymes are implicated in metabolic syndrome components, namely, obesity, hypertension, hyperglycemia, hypertriglyceridemia, and hypercholesterolemia, and this metabolic imbalance is related specifically to lipolytic and metabolic enzymes such as pancreatic lipase, *β*-glucosidase, *α*-amylase, and XO [8]. Considering all these facts and in order to prevent metabolic syndrome, searching a natural drug without undesirable side effects should be an urgent purpose. Thus, the present study is aimed at investigating if *Euphorbia cyparissias L*. (*E. cyparissias*) possesses inhibitory effects on some important digestive enzymes in addition to its possible cytotoxicity on three human cell lines.

## 2. Materials and Methods

### 2.1. Plant Material and Extraction Procedure

Fresh leaves of *E. cyparissias* (KSU No. 19378) were collected from individuals of the Al-amariah region (North of Riyadh, Saudi Arabia; 24°48′35.1^″^N 46°25′34.1^″^E, in 2007), then were identified and confirmed by Dr. Mona S. Alwahibi. After washing in distilled water, the leaves were air-dried for 3 days at room temperature (25°C) until use. A portion of ground plant material (20 g) was macerated and homogenized with 200 mL of distilled water or 70% ethanol for 24 h at ambient temperature. Thereafter, the homogenate was filtered through a Buchner funnel and centrifuged for 15 min at 5.000 rpm. The resulting supernatant was concentrated using rotary evaporator, then transferred into 100 ml beaker and left for 4 days on laboratory bench to dry properly and stored at 4°C until use.

### 2.2. Enzymes

Dromedary (DrPlA_2_-IB), porcine (PPPlA_2_-IB), and stingray group IB phospholipases (SPLA_2_-IB) were purified and prepared as described in our previous works [18, 19] and De Haas et al. [20], respectively. Dromedary (DrPL), stingray (SPL), and porcine (PPL) lipases were also purified according to protocols optimized in previous works [21–23]. The enzymes *β*-glucosidase, *α*-amylase, and XO were purchased from Sigma-Aldrich (St. Louis, MO, USA).

### 2.3. Lipolytic Enzyme Activity Inhibition

The inhibitory effect of ethanolic and aqueous extracts of *E. cyparissias* on dromedary, stingray, and porcine lipase activities were investigated at different concentrations ranging from 0 to 100 *μ*g/mL. The assay was carried out by incubating 12 IU of enzymes (50 *μ*L) with 10 *μ*L of extract. A positive control using commercial orlistat (Xenical, Hoffmann-La Roche) was run in parallel in the same conditions. Inhibition of lipase activities was expressed as the percentage of residual activity after incubation with Euphorbia extracts compared to the negative control (no enzyme added). Lipase activities were measured using tributyrin as a substrate under the optimal conditions for each enzyme, as previously described [17]. The IC_50_ values correspond to the half maximal inhibitory concentration.

### 2.4. Phospholipase Activity Inhibition

The phospholipase activities were measured based on the De Araújo and Radvanyi method [24]. The inhibitory effect of various extracts was determined using samples of dromedary, stingray, and porcine digestive group IB phospholipases (PLA2-IB). A 10 *μ*L sample of ethanolic or aqueous extract was mixed with 10 *μ*L of PLA2-GIB and preincubated for 20 min at room temperature. The mixture was added to 1 mL of the substrate, which was composed of lecithin (3.5 mM) solubilized in 100 mM NaCl, 3 mM sodium taurodeoxycholate, 10 mM CaCl_2_, and 0.055 mM red phenol at a pH of 7.6. The activity was verified by measuring spectrophotometrically the absorbance at 558 nm for 5 min. The inhibition percentage was calculated by measuring residual activity compared to negative control assay (absence of extract). The IC_50_ values were determined from the curve.

### 2.5. *α*-Amylase and *β*-Glucosidase Activity Inhibition

Alpha amylase activity was measured according to Subranian et al. [25]. A 10 *μ*L sample of *α*-amylase (3 IU) was mixed with a final concentration range from 0 to 500 *μ*g/mL of *E. cyparissias* aqueous and ethanolic extracts at 37°C for 5 min. Acarbose was used as a positive control. The residual activity was evaluated by measuring twice the absorbance at 620 nm after 8 min (A1) and 13 min (A2) after incubation with substrate (180 *μ*L of lab test diluted in water (*v*/*v*)). The reaction was carried out in a microplate (Bio-Tek ELX-800, Winooski, VT, USA). The *α*-amylase inhibition (*i*) was calculated as follows: *i* (%) = 100–(A2–A1/A2 control–A1 control) × 100, where A1 is the absorbance of the initial reading and A2 is the absorbance of the second reading.

The *β*-glucosidase activity was determined by measuring the 4-nitrophenol *α*-D′glucopyranoside (4-NPGP) product. A 180 *μ*L sample of *β*-glucosidase was preincubated for 2 min at 37°C with 20 *μ*L of *E. cyparissias* aqueous and ethanolic extracts or with a positive control (Acarbose) at a concentration range of 0 to 100 *μ*g/mL. Then, the mixture was incubated with color reagent NPGP (180 *μ*L) for more 15 min at 37°C. The colorimetric test contained 10 mM of potassium phosphate buffer, 5 mM of 4-NPGP, and 2 IU of *β*-glucosidase, at pH 6.9. The activity was evaluated by measuring absorbance at 405 nm with a microplate reader. The same equation used to measure *α*-amylase activity was used to measure that of *β*-glucosidase.

### 2.6. XO Inhibitory Activity Inhibition

According to methodology described by Bondet et al. [26], XO activity was determined by measuring the formation of uric acid from xanthine at 295 nm. The first reagent was a mixture of xanthine (667 mM), EDTA (0.1 mM), hydroxylamine (0.2 mM), and phosphate buffer (50 mM, pH 7.5). In each microplate well, 15 *μ*L of *E. cyparissias* aqueous or ethanolic extract (0 to 100 *μ*g/mL) and 40 *μ*L of XO were preincubated for 5 min at 37°C. After that, 95 *μ*L from the first reagent was added into each microplate well and incubated at 37°C for 30 min. Then, 150 *μ*L of uric acid reagent was added in the mixture and the absorbance was measured again. Ethanol was used as a negative control and allopurinol as a positive control. The same equation used to measure *α*-amylase and *β*-glucosidase activities was used to measure that of XO.

### 2.7. Protease Activity Inhibition

The trypsin and chymotrypsin activity inhibition was performed as described by Kunitz [27]. An aliquot of 1 mL of ethanolic or water extract was incubated for 15 min at 37°C with an equal volume of the enzyme. The mixture was then incubated for 30 min at 37°C with 2 mL of 2% casein as the substrate. The reaction was stopped by adding 2.5 mL of 5% trichloroacetic acid (TCA). The activity was determined by measuring the absorbance of the mixture at 280 nm, after 15 min of centrifugation at 15 000 rpm. Ethanol was used as a negative control and Bowman-Birk Inhibitor (BBI) as a positive control. The protease activity inhibition was also expressed as inhibition percentage, which was determined by comparing the results with those of a control experiment.

### 2.8. Antioxidant Assays

In every sample of *E. cyparissias* extract, the antioxidant activity was evaluated by using the DPPH radical scavenging and reducing power assays as previously described in the literature [28, 29]. To measure DPPH activity, a sample of 0.5 mL for each concentration was mixed with an equal volume of DPPH ethanolic solution and incubated in darkness for 1 h at ambient temperature. The activity was determined by comparing the absorbance at 519 nm of DPPH radicals to that of the control (containing all reagents except the extract).

To measure reducing power activity, extracts with concentrations ranging from 0.03 to 1 mg/mL were mixed with 1 mL of 1% potassium ferricyanide and 1 mL of 0.2 M sodium phosphate buffer (pH 6.6). After incubation for 20 min at 50°C, 1.25 mL of 20% TCA was added to the mixture and was centrifuged for 10 min at 3000 rpm. Finally, the upper layer solution (1.25 mL) was mixed with an equal volume of deionized water and 0.5 mL of 0.1% fresh ferric chloride. The absorbance of the obtained mixture was measured at 700 nm using distilled water as the blank and butylated hydroxytoluene (BHT) as a positive control.

### 2.9. Cytotoxicity Assays

In order to test the possible cytotoxic effect of the two extracts on human cell lines, the viability of human MDA-MB-231 breast cancer cells (ATTC, Manassas, VA, USA), Lovo, and HCT-116 cell lines were evaluated after incubation for 48 h with different concentrations of ethanolic and aqueous extracts from *E. cyparissias*. Cells were grown to confluence at 37°C under 5% CO_2_ in DMEM medium supplemented with 15% fetal bovine serum, 2 mM L-glutamine, 1 mg/mL D-glucose, 1% nonessential amino acids, 100 IU/mL of penicillin, and 100 *μ*g/mL of streptomycin. Solutions containing 10, 25, 50, and 50 *μ*g/mL were mixed with DMEM. A negative control was run in parallel using the same volume of saline buffer. After 24 h, MDA-MB-231, Lovo, and HCT-116 cells were fixed with 1% glutaraldehyde, stained with a solution of 0.1% crystal violet, and lysed with 1% sodium dodecyl sulfate (SDS). Absorbance was then measured at 600 nm. Experiments were carried out in triplicate. Cytotoxicity was expressed as a relative percentage of the optical density (OD) values measured in the control and those of ethanolic and aqueous extract-treated cells.

## 3. Results

### 3.1. Evaluation of *E. cyparissias* Extracts on Digestive Lipolytic Enzyme Inhibition

**Figure 1:**
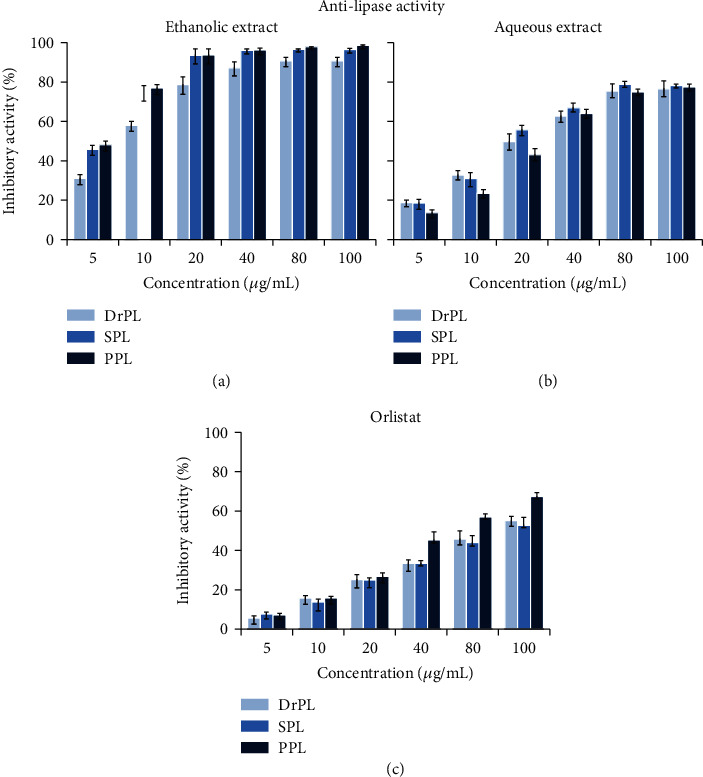
Inhibitory effect of ethanol (a) and aqueous (b) extracts of *Euphorbia cyparissias* and commercial drug orlistat (c) on the dromedary (DrPL), stingray (SPL), and porcine (PPL) lipase activities. The activities of these lipases were measured using the pH-stat technique on olive oil emulsion at optimal conditions for enzyme activity. Values represent the mean ± SD of triplicate measurements.

As shown in Figures 1(a)–1(c), the two extracts as well as the commercial inhibitor tested caused a dose-dependent inhibition at the concentrations range used. One can also note that, out of the two extracts tested, the ethanolic extract showed the most promising results in inhibiting the lipolytic activity of the three enzymes with an IC_50_ of 8.0 ± 0.4 *μ*g/mL, 2.9 ± 0.4 *μ*g/mL, and 2.3 ± 0.3 *μ*g/mL for DrPL, SPL, and PPL, respectively (Table 1). The aqueous extract inhibited the three enzymes with an IC_50_ of 23.1 ± 0.4 *μ*g/mL for DrPL, 21.0 ± 0.3 *μ*g/mL for SPL, and 27.2 ± 0.3 *μ*g/mL for PPL. Notably, the inhibitory potency of the two extracts on tested lipase activities was more pronounced than that of the commercial drug orlistat showing IC_50_ values of 97.2 ± 2.5 *μ*g/L, 109.8 ± 4.2 *μ*g/L, and 52.1 ± 2.3 *μ*g/L for DrPL, SPL, and PPL, respectively.

**Figure 2:**
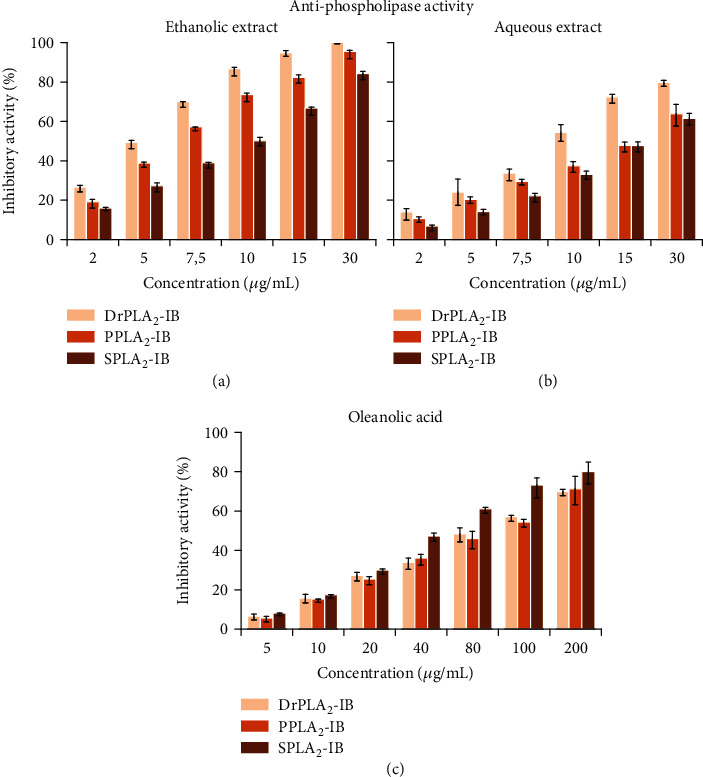
Inhibitory effect of ethanol (a) and aqueous (b) extracts of *Euphorbia cyparissias* and oleanolic acid (c) on DrPLA_2_-IB, SPLA_2_-IB, and PPLA_2_-IB phospholipase activities. The activities of these enzymes were measured using the pH-stat technique on olive oil emulsion at optimal conditions for enzyme activity. Values represent the mean ± SD of triplicate measurements.

Figure 2 shows the inhibitory effect of ethanolic and aqueous extracts from *E. cyparissias* on the three digestive phospholipases used. Interestingly, the obtained data clearly indicated that both the ethanolic and the aqueous extracts (Figures 2(a) and 2(b)) effectively suppressed activities of all phospholipases used better than the oleanolic acid used as a positive control (Figure 2(c)). The IC_50_ values presented in Table 2 showed that the ethanolic extract was about two to three times more efficient to inhibit the digestive phospholipases used than the aqueous extract. The ethanolic extract inhibited the three enzymes with an IC_50_ of 4.2 ± 0.2 *μ*g/mL for DrPLA_2_-IB, 5.9 ± 0.2 *μ*g/mL for SPLA_2_-IB, and 9.6 ± 0.2 *μ*g/mL for PPLA_2_-IB.

### 3.2. Evaluation of *α*-Amylase, *β*-Glucosidase, XO, and Protease Activity Inhibition

**Figure 3:**
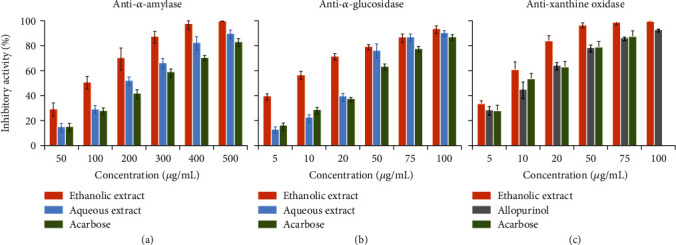
Inhibitory effect of ethanol and aqueous extracts of *Euphorbia cyparissias* on *α*-amylase (a), *β*-glucosidase (b), and antixanthine oxidase (c) activities. Values represent the mean ± SD of triplicate measurements.

As shown in Figure 3, most of the enzymes tested including *α*-amylase, *β*-glucosidase, and XO were inhibited in a dose-dependent manner after incubation with ethanolic and aqueous extracts of *E. cyparissias* (Figures 3(a)–3(c)). The values of IC_50_ presented in Table 3 allowed us to quantify more precisely the inhibitory effect of the two extracts on enzyme activities compared to those of the positive controls. Ethanolic extract presented the most promising results in inhibiting *α*-amylase, *β*-glucosidase, and XO, with IC_50_ values of 98.8 ± 8.6 *μ*g/mL, 8.1 ± 0.3 *μ*g/mL, and 7.2 ± 0.7 *μ*g/mL, respectively, followed by aqueous extract and positive controls.

**Figure 4:**
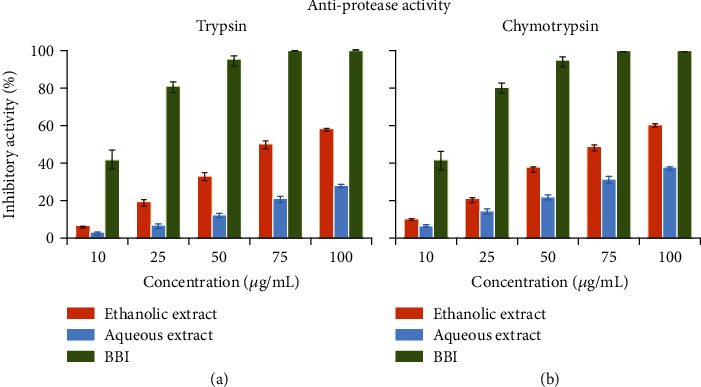
Inhibitory effect of ethanol aqueous extracts of *Euphorbia cyparissias* on trypsin (a) and chymotrypsin (b) activities. Each value is the mean of triplicate measurements ± SD.

Data presented in Figure 4 demonstrated that the inhibition of trypsin (Figure 4(a)) and chymotrypsin (Figure 4(b)) by ethanolic or aqueous extracts is limited, and complete inhibition was not achieved at the concentrations used (0 to 100 *μ*g/mL) compared to positive control. This finding is supported by the IC_50_ values presented in Table 4. BBI had the higher inhibitory effect on trypsin activity with an IC_50_ value of 11.5 ± 0.4 *μ*g/mL, followed by ethanolic extract with an IC_50_ of 84.1 ± 2.1 *μ*g/mL and by aqueous extract with an IC_50_ higher than 100 *μ*g/mL. The same result was obtained with chymotrypsin, which lost its IC_50_ at a concentration of 10.8 ± 0.4 *μ*g/mL compared to 77.5 ± 1.1 *μ*g/mL for ethanolic extract and a value higher than 100 *μ*g/mL for aqueous extract.

### 3.3. Antioxidant Activity of Ethanolic and Aqueous Extracts of *E. cyparissias*

**Figure 5:**
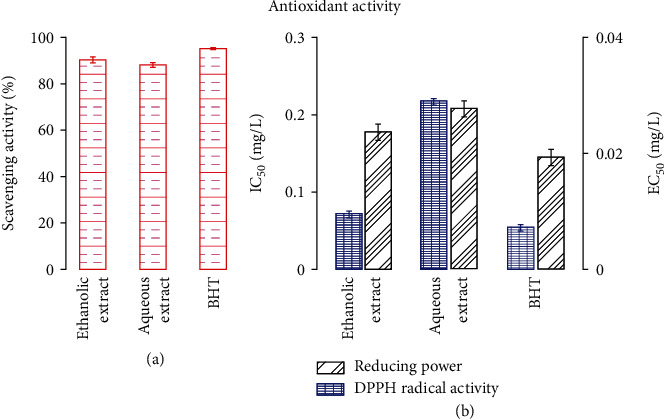
Antioxidant activities of ethanol and aqueous extracts tested with two methods: scavenging activity (a) and DPPH radical and reducing power activities (b). For each method, butylated hydroxytoluene (BHT) was used as standard, under the same conditions. Each value is the mean of triplicate measurements ± SD. ^∗^IC_50_ (mg/L): the concentration at which 50% of the activity is inhibited. ^∗^EC50 (mg/L): the effective concentration at which the absorbance reaches 0.5.

The antiradical activities of different concentration of the two extracts were determined using DPPH free radical assay, and results were compared to those of the BHT, which was used as a positive control (Figure 5(a)). The activities of the two extracts were expressed as the mean of IC_50_ (mg/mL) values (Figure 5(b)). The scavenging activities of the two extracts as well as of the BHT were concentration dependent. Results showed that, at a concentration of 1 mg/mL, the ethanolic and aqueous extracts presented high scavenging activities, with values of 90.6 ± 1.2% and 88.2 ± 1.0%, respectively, which were close to that of BHT, with 95.3 ± 0.4%. This finding was confirmed by the IC_50_ values shown in Figure 5(b), which presents an IC_50_ of ~0.073 mg/mL, ~0.22 mg/mL, and~0.054 mg/L for ethanolic extract, aqueous extract, and BHT, respectively.

### 3.4. Cytotoxicity of Ethanolic and Aqueous Extracts of *E. cyparissias*

**Figure 6:**
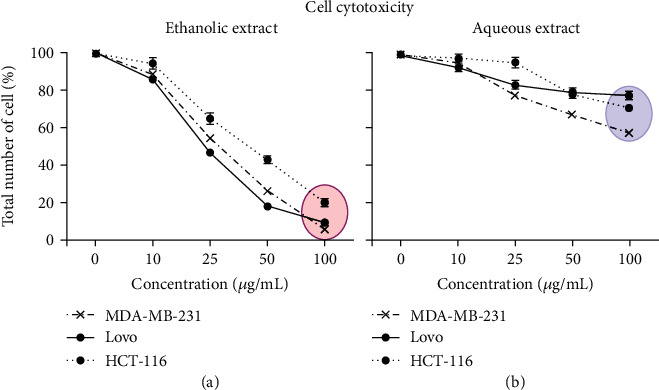
Cytotoxic effect of ethanol (a) and aqueous (b) extracts upon MDA-MB-231, Lovo, and HCT-116 cell lines. Experiments were performed in triplicate, and the results are expressed as a relative percentage of the optical density (OD) values measured in the control (untreated cells) and in the ethanolic or aqueous extract-treated cells. Results are reported as the means ± SD of triplicate measurements.

The cytotoxicity of *E. cyparissias* extracts in MDA-MB-231, Lovo, and HCT-116 cells was evaluated by measuring the cell density in the presence of increasing concentrations of extracts (0-100 *μ*g/mL) (Figures 6(a) and 6(b)). This concentration range is the same with that used in previous experiments aiming to test their inhibitory effect on digestive enzymes. Results showed that there is more cell death in the presence of the ethanolic extract than in the presence of the aqueous extract particularly at a concentration of 100 *μ*g/mL for the three type of cells used (Table 5). However, at a lower concentration, between 10 and 25 *μ*g/mL, one can note that the three cell lines used are more resistant to the two extracts than with higher concentrations. No significant decrease in the cell number was observed in the presence of the two extracts at a concentration of 10 *μ*g/mL. An average of about 55% of the total cell number was viable and resistant in the presence of 25 *μ*g/mL of the ethanolic extract. In contrast, 100% of the cells were viable in the presence of the aqueous extract.

Table 6 shows a comparison of IC_50_ average values (*μ*g/mL) calculated from individual IC_50_ values for each enzyme families presented previously. As shown in this table, most of the mean values of IC_50_ for ethanolic and aqueous extracts on all digestive enzymes used are lower than the lower cytotoxic concentration that caused 50% of cell death (30 *μ*g/mL for ethanolic extract and >100 *μ*g/mL for aqueous extract).

## 4. Discussion

According to the National Health and Nutrition Examination Surveys (NHANES, 2009-2010), approximately 69% of US adults are overweight or obese, with more than 78 million adult Americans considered obese (U.S. Department of Health and Human Services (HHS), National Institutes of Health (NIH), and National Heart, Lung, and Blood Institute (NHLBI)) [30]. It is safe to assume that obesity is a public health problem. One of the most promising targets to regulate and prevent obesity is through the inhibition of the digestion and absorption of dietary fat [31]. Pancreatic lipases are the targeted enzymes because of their role in the dietary lipids' digestion. In this study, three digestive lipases, which are DrPL, SPL, and PPL, had their activities evaluated in the presence of different concentrations (0-100 *μ*g/mL) of aqueous and ethanolic extracts of *E. cyparissias* or orlistat, a potent inhibitor of gastric and pancreatic lipases and among the most used drugs in obesity, and possibly diabetes, treatment [31, 32]. It is noteworthy that the two extracts presented a stronger inhibition effect of activities of the three lipases tested than that of orlistat at all concentrations used (Figure 1). Interestingly, *E. cyparissias* extracts and more specifically ethanolic extract (IC_50_ of 2.3 ± 0.3 *μ*g/mL) was found to be more effective in inhibiting the catalytic activity of PPL than previously reported plant extracts such as common plant sterols sitosterol and stigmasterol [33–37] and positive controls such as orlistat (IC_50_ of 12.3 ± 0.1 *μ*g/mL) [34], curcumin (IC_50_ of 13.3 ± 0.7 *μ*g/mL) [36], and quercetin (IC_50_ of 18.6 ± 0.8 *μ*g/mL) [36]. This result is valid for lipases tested (Table 6).

The group IB phospholipase A_2_ gene has previously been associated to human obesity and diabetes [38] because of the catalytic activity of its encoded enzyme, which liberates lysophospholipids. The absorption of these lysophospholipids promotes insulin resistance and tissue lipid deposition [39]. It has also been reported that group IB phospholipase A_2_ deficiency protects against high-carbohydrate diet-induced hyperlipidemia and obesity in mice [40]. Remarkably, the secretion of this enzyme predominantly during the gastrointestinal digestion suggests that oral administration of inhibitors may be a viable option for a potential therapy to suppress obesity and diabetes [41], hence the importance to discover new natural group IB PLA_2_ inhibitors.

Data of the present study clearly indicate the efficiency of *E. cyparissias* extracts even at low concentrations to inhibit digestive phospholipase activities (Figure 2, Table 6), such as the case of lipase (Figure 1), which may confirm their potential use as a natural drug against obesity and diabetes.

Metabolic syndrome is one of the greatest threats to global health, especially because it increases the probability of developing cardiovascular diseases and type 2 diabetes, and includes conditions such as obesity, atherosclerosis, and hypertension, or a combination of these metabolic abnormalities [42, 43]. People with syndrome should consume limited quantities of carbohydrates to maintain a balanced metabolism, which makes the inhibition of dietary carbohydrate digestion a logical target for pharmacological intervention, to reduce energy intake through gastrointestinal mechanisms. One of the effective solutions is to delay the glucose absorption through inhibition of key carbohydrate producing digestive enzymes. The major source of carbohydrates in the diet comes from starch, which is firstly hydrolyzed in the mouth by salivary amylase and thereafter by pancreatic *α*-amylase, producing disaccharides, which will be broken down to glucose by *β*-glucosidase in the intestine. Another biocatalyst, which is not considered a digestive enzyme but plays an important role in various forms of vascular injuries, is XO. It has previously been reported that the inhibition of the enzyme activity may exert beneficial effects on impaired vascular function associated or not with hypercholesterolemia and diabetes [44]. Thus, our purpose is to find a new natural, plant-based drug that could at the same time block XO activity and retard glucose absorption through the inhibition of carbohydrate hydrolyzing enzymes such as *α*-amylase and *β*-glucosidase to prevent metabolic syndrome.

As shown in Figure 3, the inhibitory effect of ethanolic extract is found to be ~2 to 3 times higher than that obtained with aqueous extract which could be related to elevated phenolic and flavonoids content of ethanolic fractions of *E. cyparissias* that inhibit enzyme activities more specifically compared to aqueous extract, as previously described in the literature [45]. Furthermore, such as the case of digestive lipolytic enzymes (lipases and phospholipases, Table 6), the inhibitory effect of the two extracts was more pronounced than those obtained with positive controls, which were acarbose for *α*-amylase and *β*-glucosidase and allopurinol for XO.

Unlike with lipids and carbohydrates, the benefits of inhibiting protein digestion are not clear-cut even though it may reduce calorie intake because of the suppression of digestive proteolytic activities during gastrointestinal transit. Digestion of proteins is catalyzed by a synergistic action of different digestive proteases which are secreted in the stomach (like endoproteinases), but mostly in the pancreas (trypsin, chymotrypsin, and elastase). Other proteases such as carboxypeptidase and aminopeptidase are also involved in protein digestion reactions [46].

In this study, we have selected the two pancreatic digestive proteases trypsin and chymotrypsin to observe their behavior in the presence of different concentrations of ethanolic and aqueous extracts of *E. cyparissias* (Figures 4(a) and 4(b)). The inhibitory effect of the two extracts was compared to that of the positive control with BBI [47]. These results were in line with those obtained previously by Huang et al. [48] who demonstrate that the phenolic compounds partially inhibit trypsin and a residual activity of trypsin was always maintained at 32%.

Antioxidant activities of both ethanolic and aqueous extracts were also investigated, and obtained results were compared to those of the BHT, which was used as a positive control **(**Figure 5**)**. One can notice from this figure that the two extracts had higher reducing power than that of BHT. The antioxidant activities of the two extracts could be related to the phenolic compounds produced by *E. cyparissias*, which is supported by several reports demonstrating a significant relationship between the phenolic compounds and the antioxidant activity [49, 50].

In order to study whether ethanolic and aqueous extracts of *E. cyparissias* were able to produce inhibitory or lethal effect on human cell lines, the cytotoxicity of *E. cyparissias* extracts in MDA-MB-231, Lovo, and HCT-116 cells was evaluated (Figure 6). Recorded data support our starting hypothesis of a viable use of *E. cyparissias* extracts as a therapeutic drug for metabolic syndrome or other related disease. *In vivo* and clinical studies are needed to support the results obtained in this study.

## 5. Conclusions

The results of this study indicate that E. *cyparissias* is a good source of inhibitors for various digestive enzymes (such as *α*-amylase and *β*-glucosidase, lipases, and phospholipases) and has considerable antioxidant properties. In addition, the ethanolic extract was found to present a higher inhibition activity when compared to the aqueous extract and all positive or commercial inhibitors used. Therefore, this new natural plant-based drug may be a promising alternative to prevent and treat metabolic syndrome.

## Figures and Tables

**Table 1:** Comparison of the IC_50_ values of the two extracts from *Euphorbia cyparissias* and orlistat measured using DrPL, SPL, and PPL.

	IC_50_ (*μ*g/mL)		
	DrPL	SPL	PPL
Ethanolic extract	8.0 ± 0.4	2.9 ± 0.4	2.3 ± 0.3
Aqueous extract	23.1 ± 0.4	21.0 ± 0.3	27.2 ± 2.3
Orlistat	97.2 ± 2.5	109.8 ± 4.2	52.1 ± 2.3

IC_50_ (mg/L): the concentration at which 50% of the activity is inhibited.

**Table 2:** Comparison of the IC_50_ values of the two extracts from *Euphorbia cyparissias* to commercial orlistat using DrPLA_2_-IB, SPLA_2_-IB, and PPLA_2_-IB phospholipases.

	IC_50_ (*μ*g/mL)		
	DrPLA_2_-IB	SPLA_2_-IB	PPLA_2_-IB
Ethanolic extract	4.2 ± 0.2	5.9 ± 0.2	9.6 ± 0.2
Aqueous extract	9.8 ± 0.6	18.1 ± 0.4	20.7 ± 0.5
Oleanolic acid	79.3 ± 3.5	82.8 ± 5.7	46.1 ± 0.7

IC_50_ (mg/L): the concentration at which 50% of the activity is inhibited.

**Table 3:** Comparison of the IC_50_ values of the two extracts from *Euphorbia cyparissias* to commercial acarbose and allopurinol using *α*-amylase, *β*-glucosidase, and anti-xanthine oxidase.

	IC_50_ (*μ*g/mL)		
	*α*-Amylase	*β*-Glucosidase	*α*-Xanthine oxidase
Ethanolic extract	98.8 ± 8.6	8.1 ± 0.3	7.2 ± 0.7
Aqueous extract	168.9 ± 5.5	23.5 ± 0.5	12.9 ± 0.6
Acarbose	204.1 ± 3.5	26.2 ± 2.5	—
Allopurinol	—	—	11.8 ± 0.4

IC_50_ (mg/L): the concentration at which 50% of the activity is inhibited.

**Table 4:** Comparison of the IC_50_ values of the two extracts from *Euphorbia cyparissias* to commercial acarbose and allopurinol.

	IC_50_ (*μ*g/L)	
	Trypsin	Chymotrypsin
Ethanolic extract	84.1 ± 2.1	77.5 ± 1.1
Aqueous extract	>100	>100
BBI	11.5 ± 0.4	10.8 ± 0.4

IC_50_ (mg/L): the concentration at which 50% of the activity is inhibited.

**Table 5:** Inhibitory effect of ethanol aqueous extracts of *Euphorbia cyparissias* on density of MDA-MB-231, Lovo, and HCT-116 cells compared to untreated cells at a concentration of 100 *μ*g/L.

	Cell viability (%)	
	Ethanolic extract	Aqueous extract
MDA-MB-231	5.5 ± 0.7	57.5 ± 0.7
Lovo	10.0 ± 1.4	77.0 ± 2.8
HCT-116	20.5 ± 2.1	71.0 ± 2.8

**Table 6:** Comparison of the average IC_50_ of *E. cyparissias* extracts on the studied enzymes.

	Average IC_50_ (*μ*g/mL)	
	Ethanolic extract	Aqueous extract
Lipases	4.4 ± 0.3	23.7 ± 1.3
Phospholipases	6.5 ± 0.2	16.2 ± 0.4
*α*-Amylase	98.8 ± 8.6	168.9 ± 5.5
*β*-Glucosidase	8.1 ± 0.3	23.5 ± 0.5
Xanthine oxidase	7.2 ± 0.7	12.9 ± 0.6
Proteases	80.8 ± 1.6	>100
Cytotoxicity	30 ± 1.4	>100

Grey highlighted values correspond to those of IC_50_ (*μ*g/mL) that exceed lethal concentration of 50% cell death in the cell lines used.

## Data Availability

The data used to support the findings of this study are available from the corresponding author upon request.
